# Automated MRI Field of View Prescription from Region of Interest Prediction by Intra-Stack Attention Neural Network

**DOI:** 10.3390/bioengineering10010092

**Published:** 2023-01-10

**Authors:** Ke Lei, Ali B. Syed, Xucheng Zhu, John M. Pauly, Shreyas V. Vasanawala

**Affiliations:** 1 Electrical Engineering Department, Stanford University, Stanford, CA 94305, USA; 2 Radiology Department, Stanford University, Stanford, CA 94305, USA; 3 GE Healthcare, Menlo Park, CA 94025, USA

**Keywords:** deep learning, CNN, scan prescription, MRI, region of interest, field of view

## Abstract

Manual prescription of the field of view (FOV) by MRI technologists is variable and prolongs the scanning process. Often, the FOV is too large or crops critical anatomy. We propose a deep learning framework, trained by radiologists’ supervision, for automating FOV prescription. An intra-stack shared feature extraction network and an attention network are used to process a stack of 2D image inputs to generate scalars defining the location of a rectangular region of interest (ROI). The attention mechanism is used to make the model focus on a small number of informative slices in a stack. Then, the smallest FOV that makes the neural network predicted ROI free of aliasing is calculated by an algebraic operation derived from MR sampling theory. The framework’s performance is examined quantitatively with intersection over union (IoU) and pixel error on position and qualitatively with a reader study. The proposed model achieves an average IoU of 0.867 and an average ROI position error of 9.06 out of 512 pixels on 80 test cases, significantly better than two baseline models and not significantly different from a radiologist. Finally, the FOV given by the proposed framework achieves an acceptance rate of 92% from an experienced radiologist.

## 1. Introduction

**Figure 1:**
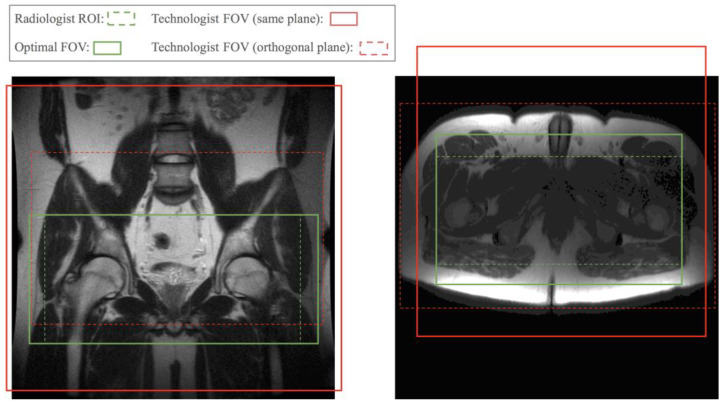
Technologist-prescribed FOV, and slice range for the orthogonal plane, compared with optimal FOV derived from radiologist’s ROI. Red solid boxes represent the FOV prescribed for the same plane as the localizer image. Red dashed boxes indicate the slice range and left–right FOV prescribed for the plane orthogonal to that of the localizer image, i.e., axial plane for the coronal localizer and vice versa. The red dashed box in the left sample truncated the bottom part of the ROI. The other three red boxes are much larger than needed.

At the beginning of clinical MRI exams, a set of localizer images with low spatial resolution and a large field of view (FOV) is collected to help define more precise imaging regions for the following diagnostic image acquisitions. MRI technologists select a FOV on the localizer images to plan the next scan. This manual step slows down the scanning workflow, prolonging the overall exam time, and influences image quality. Technologists are often not fully informed of the radiologist’s requirements for the region of interest (ROI). Figure 1 shows two examples of FOVs prescribed by a technologist compared to a radiologist’s required FOV. Poor FOV assignments may lead to images with relevant anatomy truncated and a non-diagnostic exam. A conservatively assigned large FOV costs scan time or resolution. Therefore, we propose automating the FOV assignment with models trained by radiologists’ ROI labels to obtain near optimal FOVs and streamline scans after the initial localizer.

Studies on brain [1,2,3] and knee [4] MR images have shown that, for longitudinal studies where precise reproducibility of the scan prescription is important, automatic methods achieve less variance than manual prescriptions. One study [5] presents a brain volume of interest prediction by a registration method that takes nearly one minute. The studies in [6,7] present and evaluate an automatic prescription method for knee images based on an anatomical landmark predicted by an active shape model [8] and that takes over ten seconds for inference. Deep learning-based methods have fast inferences, commonly under one second, and have become popular for organ segmentation tasks. Works on liver [9,10], spleen [11,12], and kidney [13,14] segmentations can be combined to obtain a single rectangular ROI for the abdomen. However, besides the excess number of models needed, not all ROIs can be defined completely from the edges of organs. 

**Figure 2:**
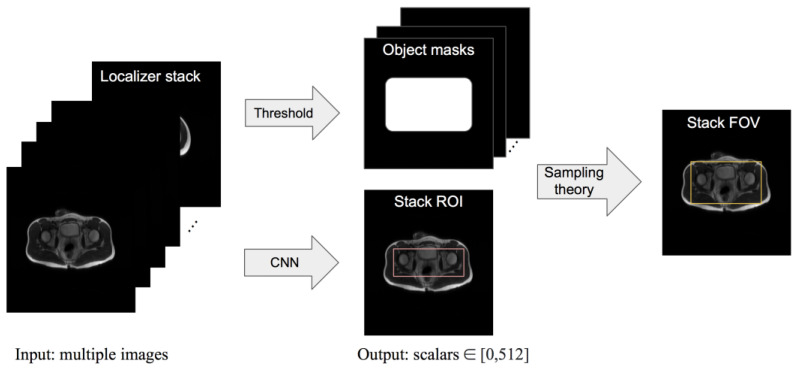
Overview of the proposed framework pipeline. First, a CNN-based model is trained to predict an ROI for a localizer stack. Then, slice-wise simplified rectangular masks of the object are obtained by setting a threshold on the row or column sums of the pixel values. The final output is the smallest FOV that places aliasing copies of the object masks outside the predicted ROI.

We present a framework where the input is a set of localizer images, the output is a rectangular ROI predicted by a convolutional neural network (CNN)-based model, and an FOV is then derived from the ROI according to MRI sampling theory. The high-level workflow is illustrated in Figure 2. We compare our model’s ROI prediction to that given by two baseline models and by an experienced radiologist. We compare our model’s FOV selection with that of a technologist and check the clinical acceptability rate of the FOV given by our framework. The purpose of this study is to construct a framework for automating FOV assignment and examine its suitability for clinical application.

## 2. Materials and Methods

### 2.1. Data Curation 

With IRB approval and informed consent/assent, we gathered all axial and coronal single-shot fast spin echo 3-plane localizer raw data from pelvic or abdominal scans of pediatric patients referred for clinical exams over a four-year period. Sex and age information was not available at the data curation stage, but a broad range of body sizes could be observed from the anonymized image samples. We then reconstructed the k-space data using Autocalibrating Reconstruction for Cartesian imaging (ARC [15]). There were 284 pelvic and 311 abdominal cases, each containing axial, coronal, and sagittal view slices. We chose the axial and coronal slices in each localizer so that a volumetric FOV could be defined. The number of slices in a localizer stack ranged from 1 to 40. 

Our study uses pediatric data because there is higher variation in body shape from pediatric patients than from adult patients, making the application more challenging. We chose pelvic and abdominal FOV assignments because these are normally based on multiple anatomies and are not organ segmentation problems. 

For a given localizer series, one of two radiologists was asked to draw a rectangular box on each, representing the desired ROI of subsequent acquisitions. The coordinates of the box’s four corners were recorded as the label for each stack. The coronal training sets were labeled by radiologist A (twenty years of MRI experience); the axial training sets were labeled by radiologist B (five years of MRI experience). The test sets were labeled by both radiologists independently. The pelvic ROI was labeled based on hip exams, ensuring coverage of the trochanters, symphysis, and hamstring origins. The abdominal ROI was labeled to tightly cover the liver, spleen, and kidneys. In abnormal cases, such as peripancreatic fluid collections or kidney transplants in the pelvis, those regions were also covered. Subcutaneous fat was included for coronal scans and excluded for axial scans because the left–right direction is the frequency encoding direction for the axial plane and the phase encoding direction for the coronal plane. The frequency encoding direction in MRI is oversampled, so there is no risk of the subcutaneous fat aliasing.

### 2.2. Data Augmentation 

To augment the training dataset, we first created a horizontally flipped copy of all original image stacks. Then, during training, all image stacks were cyclic shifted along the width and height dimensions for an integer number of pixels randomly chosen from sets, i.e., {−10, −5, 0, 5, 10} and {−20, −10, 0, 10, 20}, respectively. The boundary labels were adjusted accordingly for the augmentation samples. 

The input and output of all neural network models in this work are a stack of 2D images and two scalars, respectively. We independently trained two instances of each model to output a pair of scalars in the range of 0 and 512, representing the top and bottom or left and right boundaries of the ROI box. We used the mean squared error as the training loss for all models. We trained two instances of each model to predict the left–right and top–bottom boundaries independently because, empirically, this results in a better performance than using one or four instances. 

### 2.3. Two Baseline Models 

We first present two standard CNN models on end-to-end ROI regression from stacks of images. 

**Figure 3:**
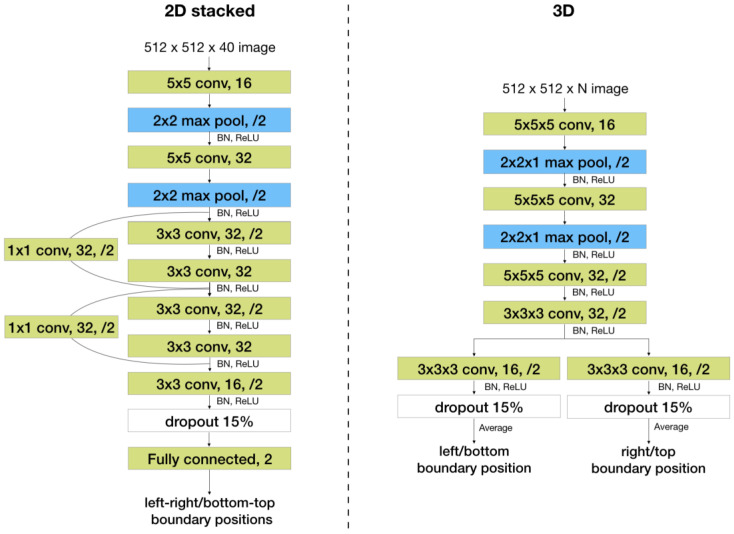
Baseline network architectures of the 2D stacked baseline model (**left**) and the 3D baseline model (**right**). For example, “3 × 3 conv, 32,/2” represents a convolutional layer with 32 kernels of size 3 × 3 and using a stride of 2. BN stands for batch normalization. “Fully connected, 2” represents a fully connected layer with two output nodes.

The first one is a 2D convolutional network with a residual block [16] and a fully connected layer, shown in Figure 3, referred to as the “2D stacked” model. Slices in a localizer are stacked on the channel dimension. Input to the network is a tensor whose height and width are equal to those of each slice and whose channel length is equal to the number of slices. The channel length of the inputs to this model must be constant, so all localizer stacks are zero padded to have a channel length equal to the maximum number of slices (i.e., 40) per stack. 

The second one is a 3D fully convolutional network, shown in Figure 3, referred to as the “3D” model. This model can take inputs with a varying number of slices if it is fully convolutional. However, it takes more runtime and memory than the 2D model. 

### 2.4. Shared 2D Feature Extractor with Attention 

To obtain the advantages of both networks above, we propose using a single-channel-input 2D CNN as the feature extractor shared among all slices and then regressing from a linear combination of the extracted features. This architecture allows for a flexible stack size as with the 3D model and has less parameters than even the 2D stacked model. 

Furthermore, to obtain an informative combination of slice features, we propose using an attention network to discriminatively weigh the features. As a localizer stack has up to 40 slices, many of them do not contain relevant anatomy for determining the ROI. A two-layer attention network is shared across all slices and trained implicitly within the whole end-to-end network. It takes an output of the feature extractor as its input and then outputs a scalar representing the importance of the corresponding slice. The resulting scalars from all slices are then passed through a SoftMax layer, defined by Equation (1), to obtain weights for the extracted features.

(1) $$\alpha_{i}=\frac{e^{z_{i}}}{\sum_{j=1}^{N}e^{z_{j}}},$$

where $z_{i}=\mathrm{Attention}\left(h_{i}\right)$. Then, the combined feature of the whole stack of *N* slices is obtained as follows:

(2) $$h_{\mathrm{combined}}=\sum_{i=1}^{N}\alpha_{i}h_{i}.$$

**Figure 4:**
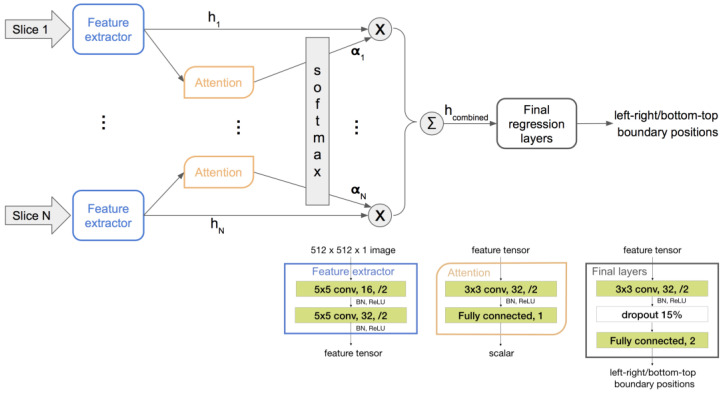
Network flow (top half) and architectures (bottom right corner) of the proposed framework, where x represents element-wise multiplication and Σ represents summation, α represents scalars that sum to one, and h represents image features. The number following “Fully connected” represents the number of output nodes of that layer. There is only one instance of the feature extractor and the attention network, and they are shared across all slices.

The weighted mean of the slice features has a fixed shape regardless of the input stack size. It is regressed to the final positional scalar outputs through a few more convolutional layers and a fully connected layer. The proposed architecture and training flow are illustrated in Figure 4. 

All neural networks are trained with an ℓ_2_ loss on the boundary positions. 

### 2.5. ROI to Smallest FOV 

The final goal of this work was to assign the smallest FOV that ensures no aliasing in the predicted ROI. We determined this FOV from a simplified model of the imaging object and the physics of MRI sampling. 

First, we found the first and last row and column of an image where the pixel value sum surpasses a threshold. These four lines constitute a rectangular mask of the object in that slice. For simplicity, we assumed that every pixel in this mask has a non-negligible signal, i.e., this mask represents the object. 

**Figure 5:**
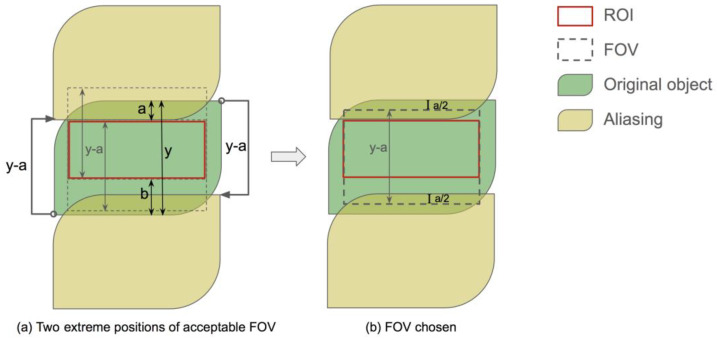
Determination of the smallest FOV in the phase encoding direction (i.e., up-down direction in the figure) to achieve an aliasing-free ROI. The object is aliased to integer numbers of FOV away from the original position, and two of the closest copies are shown in the figure. Given *a* ≤ *b*, the smallest FOV width to keep the ROI alias-free is *y* − *a*. The position of the FOV does not affect the alias-free region, but we need the ROI to be in the FOV. (**a**) shows two FOVs at the extreme positions to fully include the ROI, and everything in between is acceptable. The position we used for this work is shown in (**b**).

In the phase encoding direction, two aliasing copies came from the original object shifted for the FOV length each way, as illustrated by Figure 5a. The smallest FOV should place the aliasing copy right outside the boundary of the ROI that is closer to that of the object mask. Let the width of the object mask be *y* and the distances between the ROI and FOV boundaries on two sides be *a* and *b*, respectively, where *a* ≤ *b*. Then, the smallest FOV width is *y* − *a*. This FOV width leads to an alias-free region of width *y* − 2*a* at the center of the object mask, regardless of the FOV position. Since *y* − 2*a* ≥ *y* − *a* − *b* for the ROI width, there are a range of positions where the FOV box can be to include the ROI. We chose to put one of the FOV boundaries at the midpoint between the object mask and the ROI, $y-\frac{a}{2}$, to center the alias-free region, as shown in Figure 5b. 

In the readout direction, aliasing was easily avoided due to a high sampling rate, and the FOV was set by the anti-aliasing filter. We set the FOV boundaries equal to those two of the ROI in this direction. 

Finally, the union of the FOV from all slices in the localizer was used for the upcoming scan. 

### 2.6. Evaluation Measures 

We used two quantitative metrics to evaluate the performance of the ROI prediction, given that we had ground truth labels for the ROI. We performed a qualitative reader study for the FOV assignment, which is the final output of the proposed framework. 

Area intersection over union (IoU) and position error were used to measure the difference between two ROIs. The position error is the distance between two boundary lines measured in pixels and averaged across the four sides of an ROI box. Labels from radiologist A were used as the ground truth for the coronal test sets; labels from radiologist B were used as the ground truth for the axial test sets. The ROIs given by the models and the other radiologist were compared to these ground truths. 

For the qualitative reader study, the aliasing-free region resulting from our model-predicted FOV was shown to radiologist A, and we asked if this aliasing-free region was clinically acceptable. 

We conducted two-tailed t-tests using SciPy 1.1.0 on the null hypothesis of the two methods having comparable position error or IoU; *p*-values of 0.05 and 0.01 were used for significance. 

## 3. Results

### 3.1. Baseline Models

The ROIs predicted by the standard 2D model on channel stacked inputs and the 3D convolutional model were evaluated by comparing to the labels given by a radiologist on four datasets: axial pelvic, coronal pelvic, axial abdominal, and coronal abdominal localizers. Each test dataset consisted of 20 cases chosen randomly from all cases curated for this study. 

The boundary position error and IoU from the above comparison are shown in the first two columns of Table 1 and Table 2, respectively. The 2D model achieved average position errors ranging from 12.76 to 22.60 pixels out of 512 pixels and an average IoU ranging from 0.693 to 0.846 on four datasets. The 3D model achieved average position errors ranging from 11.25 to 24.33 pixels out of 512 pixels and an average IoU ranging from 0.701 to 0.852 on four datasets. Both baseline models are significantly (*p* < 0.01) worse than the proposed model and the radiologists. Both models performed better on the abdominal dataset than on the pelvic one. Neither of the two baseline models performed consistently better than the other. However, the 3D convolution model had a four times longer inference time than the 2D convolution-based models. 

### 3.2. Shared 2D Feature Extractor with Attention 

The shared 2D feature extractor and attention model was evaluated by comparing its ROI prediction to the labels given by a radiologist on the same four test datasets as above. The difference between this model and a radiologist, shown in the third column of Table 1 and Table 2, was then compared with the difference between two radiologists, shown in the fourth column of Table 1 and Table 2. 

The proposed model achieved average boundary position errors ranging from 6.15 to 11.61 pixels out of 512 pixels and an average IoU ranging from 0.835 to 0.874 on four datasets. This performance is significantly better than both baseline models (*p* < 0.01 on ten and *p* < 0.05 on the other six out of sixteen comparisons) and comparable to that of a radiologist (*p* > 0.12 on seven out of eight comparisons). 

**Figure 6:**
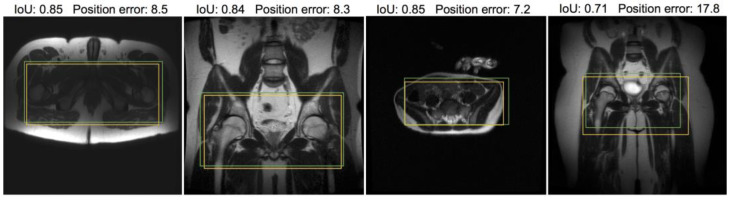
Samples from pelvic localizer test dataset annotated with intra-stack attention model-predicted ROI (yellow) and the ROI label given by radiologists (green). To help visually interpret the quantitative results, the IoU and position error for the model prediction in each example are included. Note that only one representative slice of a stack is shown, and neither ROI was determined merely from this slice. The left three samples are representative of the average model performance, while the rightmost example is a poorly performed case.

**Figure 7:**
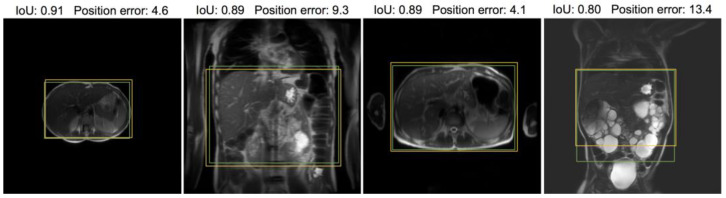
Samples from abdominal localizer test dataset annotated with intra-stack attention model-predicted ROI (yellow) and the ROI label given by radiologists (green). For visual interpretation of the quantitative results, the position error and IoU for the model prediction in each example are included. Note that only one representative slice of a stack is shown, and neither ROI was determined merely from this slice. The left three samples are representative of the average model performance, while the rightmost example is a poorly performed case. Note that the rightmost one is an extreme case of polycystic kidney disease.

Figure 6 and Figure 7 provide visual examples of the predicted ROI compared with the labeled ROI on pelvic and abdominal localizers, respectively. While the ROI is based on the whole stack, one representative slice is used for illustration. 

### 3.3. FOV Acceptability 

We presented the resulting alias-free region when using the FOV given by our framework to a radiologist and asked whether this end result is clinically acceptable, i.e., the ROI is within the alias-free region and not too much smaller than it. The radiologist was given three ratings to choose from: yes, almost, and no. In total, 40 pelvic cases and 40 abdominal cases were included in this study. Overall, 69 cases received the rating “yes”, 9 cases received the rating “almost”, and 2 cases received the rating “no”. 

We calculated the 80% confidence interval for proportions for the counts above. The frequency interval of the “yes” rating was 0.80 to 0.91, and the frequency intervals of the “yes” and “almost” ratings were 0.93 to 0.99. 

### 3.4. Inference Time and Implementation 

The average inference time for one stack of localizer images was 0.12, 0.44, and 0.12 s for the 2D stacked baseline, 3D baseline, and 2D intra-stack attention model, respectively. A batch size of one and one NVIDIA GeForce GTX 1080 GPU were used for the inference. All models were trained from random parameter initialization. The models were implemented in TensorFlow 2.0 and Keras, and the implementation code is available on GitHub*. * https://github.com/lisakelei/MRI-ROI-prediction.

## 4. Discussion

We developed a CNN-based model with intra-stack parameter sharing and attention suitable for automating the FOV prescription step in MR scans. In the FOV, the prescription given by the proposed model achieved a 92% acceptance rate (cases rated as “almost” acceptable were counted as halves) for clinical scans. In the ROI, the proposed model achieved an average IoU of 0.849 and an average position error of 10.93 out of 512 pixels on 40 pelvic scans. It achieved an average IoU of 0.885 and an average position error of 7.19 out of 512 pixels on 40 abdominal scans. This performance is significantly better than that of standard CNN models without attention or parameter sharing and is not significantly inferior to the variance between two radiologists. The model-predicted FOV is significantly more compact than the one manually prescribed by MRI technologists, which, despite being conservatively large, sometimes misses parts of interests.

We noticed that the objects in abdominal localizers appear more zoomed out than those in the pelvic localizer, and the abdominal ROI takes up a larger portion of the whole object shown in the localizers than the pelvic ROI does. This may be one reason that our framework performed slightly better on the abdominal dataset than on the pelvic dataset. This highlights that the technique used to obtain the localizers may be critical to performance. 

There is no existing work on deep learning-based end-to-end FOV box prediction to our knowledge. Our framework is at least 100 times faster than existing non-deep-learning-based methods. Its inference time is 0.12 s per stack of images on a GPU and 0.17 s per stack on a CPU, compared to 10 s or 1 min from previous works [5,6]. The existing works [1,2,3,4,5,6,7] were performed on brain or knee images and thus cannot be adapted to our pelvic and abdominal images without additional information. Therefore, in this work, the performance comparison was only performed across deep learning models. 

In addition to the target application of this work, namely automatic scan prescriptions, the ROI prediction network can be useful for image quality assessment when the FOV is larger than the ROI. Instead of assessing the whole image [17], assessing only the ROI gives more relevant and accurate evaluation results. This difference in the region of assessment is particularly influential with respect to localized artifacts that degrade image quality. 

One limitation of the presented framework is that it does not support oblique ROI and FOV prediction. Some objects in the localizer images were rotated, in which case the most efficient FOV was aligned with the object and not aligned with the localizer image. Adding support on predicting a rotated box is the next step for future work. The majority of the current framework holds, except that the neural network then needs to output five or six scalars defining a rotated rectangle instead of four scalars defining a rectangle along the coordinate axes. 

We have presented a framework for ROI and FOV prediction on pelvic and abdominal MR scans. The framework utilizes a neural network model for ROI prediction from a stack of localizers, followed by a thresholding step and an MR physics-derived algebraic conversion to an FOV. We have proposed an architecture consisting of a slice-wise shared CNN and an attention mechanism for the bounding box regression model and compared its performance with standard CNN models and a radiologist. The framework has been examined on a set of cases with large patient size/shape variation and abnormalities. It can be applied clinically to reduce the time gap between series of scans and to obtain a more efficient and precise FOV for diagnostic scans. 

## Figures and Tables

**Table 1:** Comparison of area intersection over union (IoU) averaged over cases.

Dataset	2D Stacked	3D	Intra-Stack Attention	Radiologist
Pelvis (axial)	0.751 ± 0.169 ^†^	0.701 ± 0.183 ^‡^	0.863 ± 0.106	0.874 ± 0.048
Pelvis (coronal)	0.693 ± 0.186 ^‡^	0.722 ± 0.122 ^‡^	0.835 ± 0.056	0.815 ± 0.074
Abdomen (axial)	0.846 ± 0.055 ^‡^	0.852 ± 0.051 ^‡^	0.896 ± 0.033	0.916 ± 0.022 ^†^
Abdomen (coronal)	0.833 ± 0.062	0.838 ± 0.066 ^†^	0.884 ± 0.060	0.877 ± 0.086

Three models were compared with the inter-rater variance between two radiologists across four datasets with 20 cases each. The mean ± standard deviation is reported, with significance levels noted as follows: ^†^ 0.01 ≤ *p* < 0.05 in comparison to intra-stack attention (ours), ^‡^ *p* < 0.01 in comparison to intra-stack attention.

**Table 2:** Comparison of boundary position error averaged over four sides then over cases.

Dataset	2D Stacked	3D	Intra-Stack Attention	Radiologist
Pelvis (axial)	17.13 ± 12.31 ^†^	22.98 ± 17.30 ^‡^	10.26 ± 8.61	7.02 ± 3.71
Pelvis (coronal)	22.60 ± 16.15 ^‡^	24.33 ± 20.48 ^‡^	11.61 ± 4.18	14.32 ± 5.72
Abdomen (axial)	12.76 ± 9.49 ^‡^	11.25 ± 7.96 ^‡^	6.15 ± 1.97	5.28 ± 1.52
Abdomen (coronal)	13.47 ± 8.83 ^†^	12.96 ± 8.55 ^†^	8.23 ± 4.05	9.46 ± 8.25

Three models were compared with the inter-rater variance between two radiologists across four datasets with 20 cases each. The mean ± standard deviation is reported, with significance levels noted as follows: ^†^ 0.01 ≤ *p* < 0.05 in comparison to intra-stack attention (ours), ^‡^ *p* < 0.01 in comparison to intra-stack attention.

## Data Availability

The source code for this study is available in a publicly accessible repository. The data used in this study are available on request from the corresponding author.
